# Complete Genome Sequences of Pseudomonas sp. Strains MM223 and MM227 and *Rheinheimera* sp. Strain MM224, Isolated from a Pond Edge in Bielefeld, Germany

**DOI:** 10.1128/mra.01185-22

**Published:** 2023-01-05

**Authors:** Bianca Frommer, Theresa Folchert, Anna-Lena Witschel, Mailin Reddeker, Magdalena A. R. Hauck, Nina Anderle, Victoria S. E. Schade, Laura Zagami, Marielle Rieks, Lutz Wobbe, Andrea Bräutigam, Marion Eisenhut

**Affiliations:** a Computational Biology, Faculty of Biology, Bielefeld University, Bielefeld, Germany; b Biology, Bielefeld University, Bielefeld, Germany; c Center for Biotechnology, Bielefeld University, Bielefeld, Germany; d Cluster of Excellence on Plant Sciences, Heinrich-Heine University, Düsseldorf, Germany; University of Arizona

## Abstract

Pseudomonas sp. strain MM223, Pseudomonas sp. strain MM227, and *Rheinheimera* sp. strain MM224 were isolated from a muddy soil sample from the edge of a pond. Here, we present whole-genome sequences and phylogenetic classifications for all three bacterial isolates.

## ANNOUNCEMENT

Soil quality and health are major factors for crop plant performance and thus are fundamental to world food security. The composition and diversity of soil microbial communities and their functional implications in soil ecosystem sustainability are of high interest but little understood (1). Involvement in soil health was suggested for Pseudomonas spp. because they confer suppressiveness to soilborne plant pathogens, such as the fungus Gaeumannomyces graminis var. *tritici* (2). We sequenced three bacterial isolates from a muddy field sample to enhance genomic information on bacteria belonging to soil microbial communities.

We isolated all strains from a muddy soil sample that was collected at the edge of a pond in Bielefeld, North Rhine-Westphalia, Germany (52°2′26.03184″N, 8°29′54.7296″E). The sample was taken from 5 cm beneath the ground, suspended in 0.9% (wt/vol) NaCl, filtered using a cellulose filter (pore size, 4 to 12 μm; product number 431015; Macherey-Nagel, Düren, Germany), and centrifuged. The cell pellet was resuspended in fresh 0.9% NaCl solution, and dilutions were plated on agar medium (1.5% agar, 1% soy peptone, 0.3% NaCl, 0.1% sucrose, 0.1% cellulose, 0.1% xylan, 0.1% chitin, 0.05% Tris-HCl). Colonies appeared after incubation at 30°C for 7 days. Single colonies were picked, streaked on agar medium for propagation, and used for DNA isolation with the NucleoSpin microbial DNA kit (Macherey-Nagel) with RNA digestion according to the manufacturer’s manual. Barcoding of the genomic DNA was performed using the rapid barcoding kit (SQK-RBK004; Oxford Nanopore Technologies [ONT], Oxford, UK) according to the manufacturer’s protocol. For sequencing, one R9.4.1 flow cell was run for 15 h on a GridION system, and bases were called using the super-accurate base-calling model of MinKNOW v22.05.7 (all from ONT). The sequencing reads were processed for each barcode. All programs were run with default parameters unless otherwise specified. Adapters were trimmed with Porechop v0.2.3 (3), and BLAST was used to determine the expected genome sizes (4). The genome sequence was assembled as a single contig each with Canu v2.2 (5), using a genome size of 6.2 Mbp for Pseudomonas sp. strain MM223 and a genome size of 4.6 Mbp for *Rheinheimera* sp. strain MM224 and Pseudomonas sp. strain MM227. For MM223, we obtained an additional contig for a plasmid. Assemblies were polished with Racon v1.5.0 (6), Minimap v2.24-r1122 with parameter setting –ax map-ont (7), and Medaka v1.6.0 with parameter setting –m r941_min_sup_g507 (ONT). Overlaps were trimmed with Berokka v0.2 (https://github.com/tseemann/berokka), and contigs were oriented according to *dnaA* with Circlator v1.5.5 (8). Genome completeness was examined with benchmarking universal single-copy orthologs (BUSCO) v5.4.3 with parameter setting –augustus (9). Genes were predicted with Prokka v1.14.5 (10). The organism and strain type were identified with the Type (Strain) Genome Server (TYGS) (11). Relevant statistics for the raw reads and genome and plasmid sequences are listed in Table 1.

**TABLE 1:** Sequencing and assembly statistics for Pseudomonas sp. strains MM223 and MM227 and *Rheinheimera* sp. strain MM224

Parameter	Finding for:		
	Pseudomonas sp. strain MM223	Pseudomonas sp. strain MM227	*Rheinheimera* sp. strain MM224
Raw sequencing reads			
No. of reads	48,258	123,703	151,807
Total length (bp)	447,103,666	962,244,628	961,165,805
*N*_50_ (bp)	15,314	12,688	11,173
Genome sequence			
Length (bp)	6,730,273	4,620,590	4,642,311
GC content (%)	61.95	62.22	46.43
Genome coverage (×)	66.43	208.25	207.04
Gene annotation			
Total no. of genes	6,378	4,201	4,311
No. of protein-coding genes	6,275	4,112	4,216
No. of rRNAs	22	19	15
No. of tRNAs	80	69	79
No. of transfer-messenger RNAs	1	1	1
BUSCO results (%)^*a*^			
Complete	94.6	99.2	98.1
Single copy	94.5	99.2	98.0
Duplicated	0.1	0	0.1
Fragmented	3.7	0.1	0.6
Missing	1.7	0.7	1.3
Plasmid			
Length (bp)	2,397		
No. of protein-coding genes	2		
GC content (%)	47.27		
Plasmid coverage (×)	287		

a The databases used (and the numbers of searched BUSCOs) were as follows: MM223 and MM227, pseudomonadales_odb10 (782 BUSCOs); MM224, alteromonadales_odb10 (820 BUSCOs).

**FIG 1:**
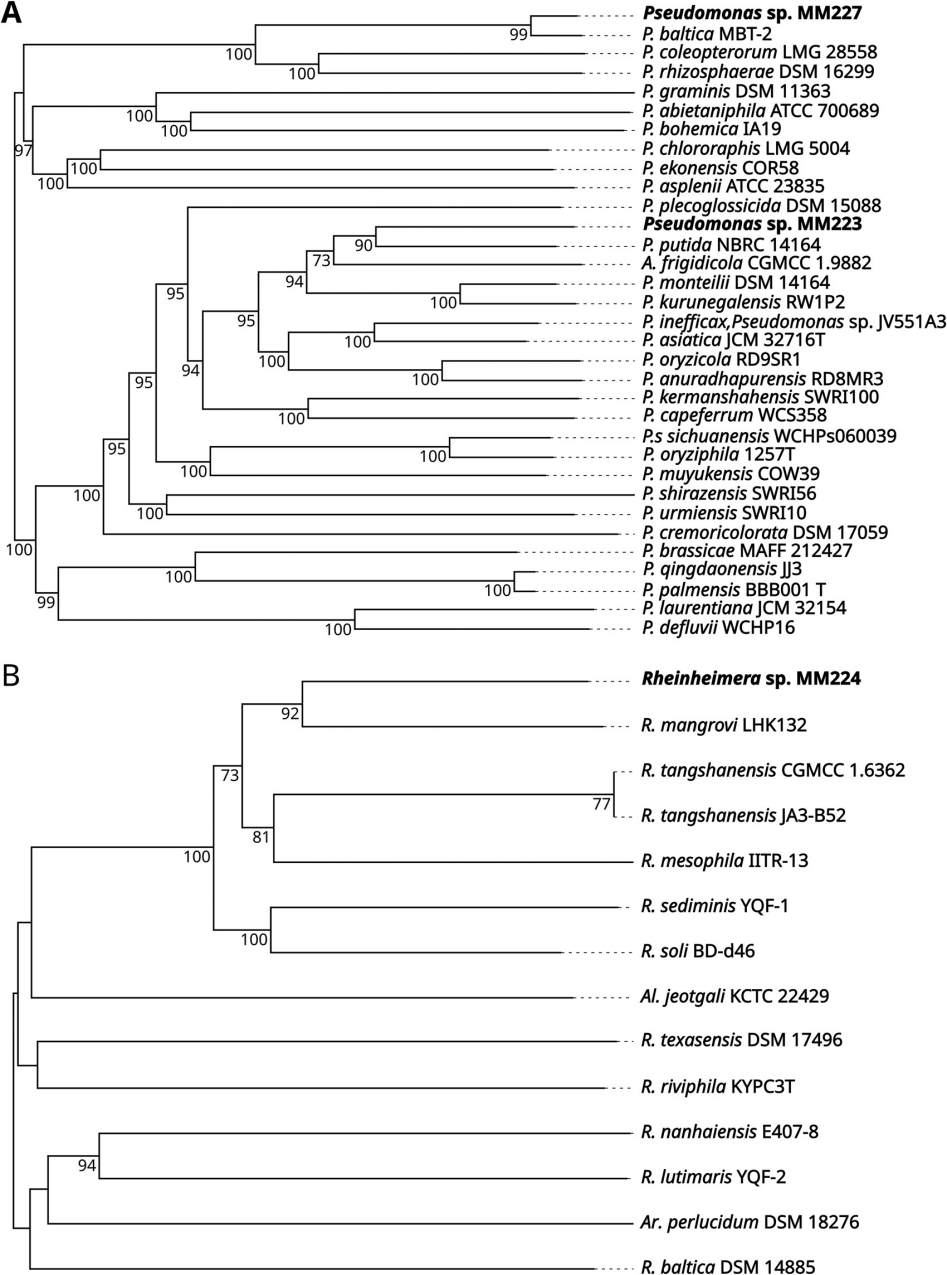
Genome BLAST distance phylogeny (GBDP) trees. Phylogenetic trees for Pseudomonas sp. strains MM223 and MM227 (A) and *Rheinheimera* sp. strain MM224 (B) were constructed from genome sequences using the TYGS pipeline (11). (A) The digital DNA-DNA hybridization (dDDH) (formula d4) similarity between MM223 and its closest relative, Pseudomonas putida NBRC 14164, is 51.2%, below the 70% species delineation threshold; therefore, Pseudomonas sp. strain MM223 is a potential new species (11). In contrast, the dDDH (formula d4) value for MM227 with Pseudomonas baltica MBT-2 is 84.4%, indicating that MM227 belongs to the species Pseudomonas baltica. (B) With a dDDH (formula d4) value of 34.7% for Rheinheimera mangrovi LHK, *Rheinheimera* sp. strain MM227 also is defined as a potential new species. The presented isolates MM223, MM224, and MM227 are highlighted in bold type. The pseudo-bootstrap support values from 100 replications are indicated at each branch point. *A*., *Arthrobacter*; *Al*., *Alishewanella*; *Ar*., *Arsukibacterium*; *P*., Pseudomonas.

The genome of Pseudomonas sp. strain MM223 has Pseudomonas putida NBRC 14164 (GenBank accession number NC_021505.1) (12) as the closest relative (Fig. 1A). The coisolated plasmid contains two open reading frames, encoding hypothetical proteins with unknown functions. The other Pseudomonas sp. strain, MM227, is closely related to Pseudomonas baltica MBT-2 (GenBank accession number GCA_014235765.1) (13) (Fig. 1A).

The third genome belongs to isolate MM224. Its closest relative is Rheinheimera mangrovi LHK132 (GenBank accession number GCA_003990335.1) (Fig. 1B).

### Data availability.

The MM223, MM224, and MM227 assemblies, gene annotations, and reads are available at GenBank/ENA under the BioProject accession number PRJEB56339. The accession numbers for MM223 are ERS13525430 (raw reads) and ERS13579685 (assembly and annotation), those for MM224 are ERS13525431 (raw reads) and ERS13579686 (assembly and annotation), and those for MM227 are ERS13525432 (raw reads) and ERS13579687 (assembly and annotation).
